# *Borealodon osedax*, a new stem mysticete (Mammalia, Cetacea) from the Oligocene of Washington State and its implications for fossil whale-fall communities

**DOI:** 10.1098/rsos.182168

**Published:** 2019-07-24

**Authors:** B. K. Shipps, Carlos Mauricio Peredo, Nicholas D. Pyenson

**Affiliations:** 1 Department of Atmospheric, Oceanic, and Earth Sciences, George Mason University, Fairfax, VA, USA; 2 Department of Paleobiology, National Museum of Natural History, Washington, DC, USA; 3 Department of Earth and Environmental Science, University of Michigan, Ann Arbor, MI, USA; 4 Department of Paleontology and Geology, Burke Museum of Natural History and Culture, Seattle, WA, USA

**Keywords:** baleen, Cetacea, Mysticeti, Oligocene, Pysht Formation

## Abstract

Baleen whales (mysticetes) lack teeth as adults and instead filter feed using keratinous baleen plates. They do not echolocate with ultrasonic frequencies like toothed whales but are instead known for infrasonic acoustics. Both baleen and infrasonic hearing are separately considered key innovations linked to their gigantism, evolutionary success and ecological diversity. The earliest mysticetes had teeth, and the phylogenetic position of many so-called toothed mysticetes remains debated, including those belonging to the nominal taxonomic groups Llanocetidae, Mammalodontidae and Aetiocetidae. Here, we report a new stem mysticete, *Borealodon osedax* gen. et sp. nov., from the Oligocene of Washington State, USA. *Borealodon* preserves multi-cusped teeth with apical wear; microCT scans of the inner ear indicate that the minimum frequency hearing limit of *Borealodon* was similar to mammalodontids. *Borealodon* is not recovered within a monophyletic Mammalodontidae nor a monophyletic Aetiocetidae; instead, it represents an unnamed lineage of stem Mysticeti, adding to the diversity of stem mysticetes, especially across the Rupelian–Chattian boundary. Furthermore, the presence of a putative chemosynthetic bivalve along with *Osedax*, a bone-boring annelid, found in association with the type specimen of *Borealodon*, offer more insights into the evolution of deep-sea whale-fall communities.

## 1. Introduction

Modern baleen whales are a clade of mammals that have evolved to include the largest vertebrates in history. Their evolutionary and ecological success is facilitated by key innovations that further aid their life in the water, such as low-frequency hearing and filter feeding with baleen plates [1]. These innovations occur prior to the origin of crown mysticetes, in the late Eocene and early Oligocene—well after the initial land–sea transition of stem cetaceans—suggesting (i) that they are not merely a function of returning to a marine environment and (ii) that they may be integral to the early success and diversification of the clade [1].

Stem baleen whales originate in the late Eocene [2,3] from toothed ancestors, and toothed, stem mysticetes persist into the Oligocene [4]. At least four distinct clades of stem, toothed mysticetes exist, each with widely disparate tooth morphologies spanning a broad range of potential ecological roles [5,6]. This dental diversity is in stark contrast to modern baleen whales, which are edentulous and feed with keratinous baleen plates. Recent work [7–9] indicates that the loss of teeth is a fundamentally decoupled evolutionary question from the origin of baleen, though they may still be linked developmentally [10]. Therefore, understanding the diversity and trends associated with the evolution of stem mysticete teeth is integral to understanding the broader teeth-to-baleen transition.

Toothed mysticetes include the late Eocene *Llanocetus* and *Mystacodon*, the early Oligocene *Coronodon*, and the Oligocene aetiocetids and mammalodontids. Each clade preserves morphological characteristics that are transitional states between stem cetaceans (so-called archaeocetes) and crown mysticetes. In particular, mammalodontids such as *Janjucetus* and *Mammalodon*, and aetiocetids such as *Aetiocetus*, *Chonecetus*, *Salishicetus* and *Fucaia* are among the last surviving toothed mysticetes. Here, we describe *Borealodon osedax* gen. et sp. nov., a stem mysticete from the Pysht Formation of Washington, USA. *Borealodon* represents an unnamed lineage of stem mysticete crown-ward of mammalodontids and stem-ward of aetiocetids. Its dental wear, low-frequency hearing capabilities and the type skeleton's association with whale-fall invertebrate taxa (notably, *Osedax*) make *Borealodon* relevant for understanding the palaeobiology and palaeoecology of early Mysticeti.

## 2. Material and methods

### 2.1. Specimen history

The holotype specimen of *B. osedax* has been figured by previous authors describing its *Osedax* borehole traces [11,12]. However, several critical discrepancies exist across these two studies, and we reconcile them here. Kiel *et al.* [11] initially reported and figured a rib fragment associated with the holotype specimen of *B. osedax* using the catalogue number USNM 539939. However, later in the same manuscript, they refer to this specimen using the catalogue number USNM 539938. In their subsequent study [12], they refer to the holotype specimen again using the catalogue number USNM 539938. This discrepancy is an error; both studies refer to the holotype specimen of *B. osedax* that represents a single individual with associated skeletal elements. Based on priority, we advocate using USNM 539939 as the single catalogue number for the type specimen of *B. osedax* and we propose suppressing the confusing secondary catalogue number USNM 539938. Additionally, the reported rib fragment is inconsistent with the rib morphology of other toothed mysticetes and we instead identify it as a fragment of mandible.

The two studies in question [11,12] figured distinct isolated elements of the type specimen of *Borealodon.* In particular, Kiel *et al.* [12] figured three isolated teeth; however, the holotype specimen preserves elements of at least seven, and as many as nine teeth, all of which are figured here. Additionally, the most complete tooth has been damaged in the interim; we confirm the unfortunate loss of the terminal tooth roots since the time when it was originally figured by Kiel *et al.* [12] (figure 2*c*).

The two studies in question present further discrepancies over the type locality of the holotype specimen. Kiel *et al.* [11] originally reported the type locality as being equivalent to LACM locality 5412, and state that it is situated ‘…west of the mouth of Murdock Creek…’. However, Kiel *et al.* [12] reported that LACM locality 5412 is ‘…on the beach terrace east of Murdock Creek…’. LACM locality 5412 is the type locality for the stem odontocete *Olympicetus avitus* [13]. However, field notes by one of the collectors (James L. Goedert) suggest that the holotype specimen of *B. osedax* is more accurately provenanced from LACM locality 5123, which is the type locality for the desmostylian *Behemotops proteus* [14]. These two localities are approximately 80 cm apart in physical space (i.e. less than 1 m apart) and they may more accurately represent a single locality. Regardless, both localities are unequivocally west of Murdock Creek, and thus, we consider the contradictory statement by Kiel *et al.* [12] to be an error.

### 2.2. Digital methods

We scanned the skull, mandible fragments, tympanic bulla, periotic and three teeth of the holotype (USNM 539939) at National Technical Systems in Belcamp, MD, USA, using their Nikon Metrology's combined 225/450 kV microfocus X-ray and computed tomography (CT) walk-in vault system. This system collected slices of CT data with a slice thickness of 0.03 mm resulting in high-quality three-dimensional (3D) renderings. DICOM files from these scans were processed in Mimics (Materialise NV, Leuven, Belgium), to create 3D models of each osteological element. The 3D models are archived at Zenodo (zenodo.org) at the following DOI:10.5281/zenodo.1340712. https://zenodo.org/record/1340712#.XLX6gC_MzOQ.

### 2.3. Phylogenetic analysis

To test the phylogenetic position of USNM 539939 relative to other stem mysticetes, we coded USNM 539939 into the recent matrix from Peredo *et al.* [8,15], a modified matrix from Boessenecker & Fordyce [16]. We chose this matrix because, following the description of *Salishicetus*, it includes the coding for the holotype species of every aetiocetid and two out of three mammalodontid species. The final matrix included 363 morphological characters and 87 operational taxonomic units. We conducted a cladistic analysis using the TNT* [17] software using unordered and equally weighted characters. We used the ‘traditional search’ option in TNT, resulting in 359 most parsimonious trees with a best score of 1595 steps. The final matrix can be found in the electronic supplementary material.

### 2.4. Specimens observed

*Aetiocetus cotylalveus* (USNM 25210), *Aetiocetus polydentatus* (cast of AMP 12 reposited at LACM), *Aetiocetus weltoni* (UCMP 122900), *Chonecetus sookensis* (NMC 12095), *Chonecetus tomitai* (cast of AMP 2 reposited at LACM)*, Chonecetus yabukii* (cast of AMP 1 reposited at LACM), *Fucaia buelli* (UWBM 84024), *Fucaia goedertorum* (LACM 131146), *Janjucetus hunderi* (cast of NMV P216929 reposited at USNM), *Mammalodon colliveri* (cast of NMV P199986 reposited at USNM) and *Salishicetus meadi* (UWBM 50004).

### 2.5. Institutional abbreviations

AMP, Ashoro Museum of Paleontology; LACM, Natural History Museum of Los Angeles County; MUGD, University of Melbourne School of Earth Sciences; NMC, National Museum of Canada; NMV, National Museums Victoria; UCMP, University of California Museum of Paleontology; UWBM, Burke Museum of Natural History; USNM, National Museum of Natural History, Smithsonian Institution.

## 3. Systematic palaeontology

Cetacea Brisson, 1762

Pelagiceti Uhen, 2008

Neoceti Fordyce & de Muizon, 2001

Mysticeti Gray, 1864

*Borealodon*, gen. nov.

**LSID**. urn:lsid:zoobank.org:pub:EDB8544C-F109-453D-BFD5-8A41CFEDE90C

**Type species:** *Borealodon osedax,* gen. et sp. nov.

**Etymology.** Boreal-, meaning of or relating to the Northern Hemisphere or north; and the Latin ‘-*odon’* for tooth. The genus name calls to the diversity of toothed mysticetes from the North Pacific.

**Diagnosis.** As for type and only species.

*Borealodon*, sp. nov. (figures 1–10).

**Figure 1.**
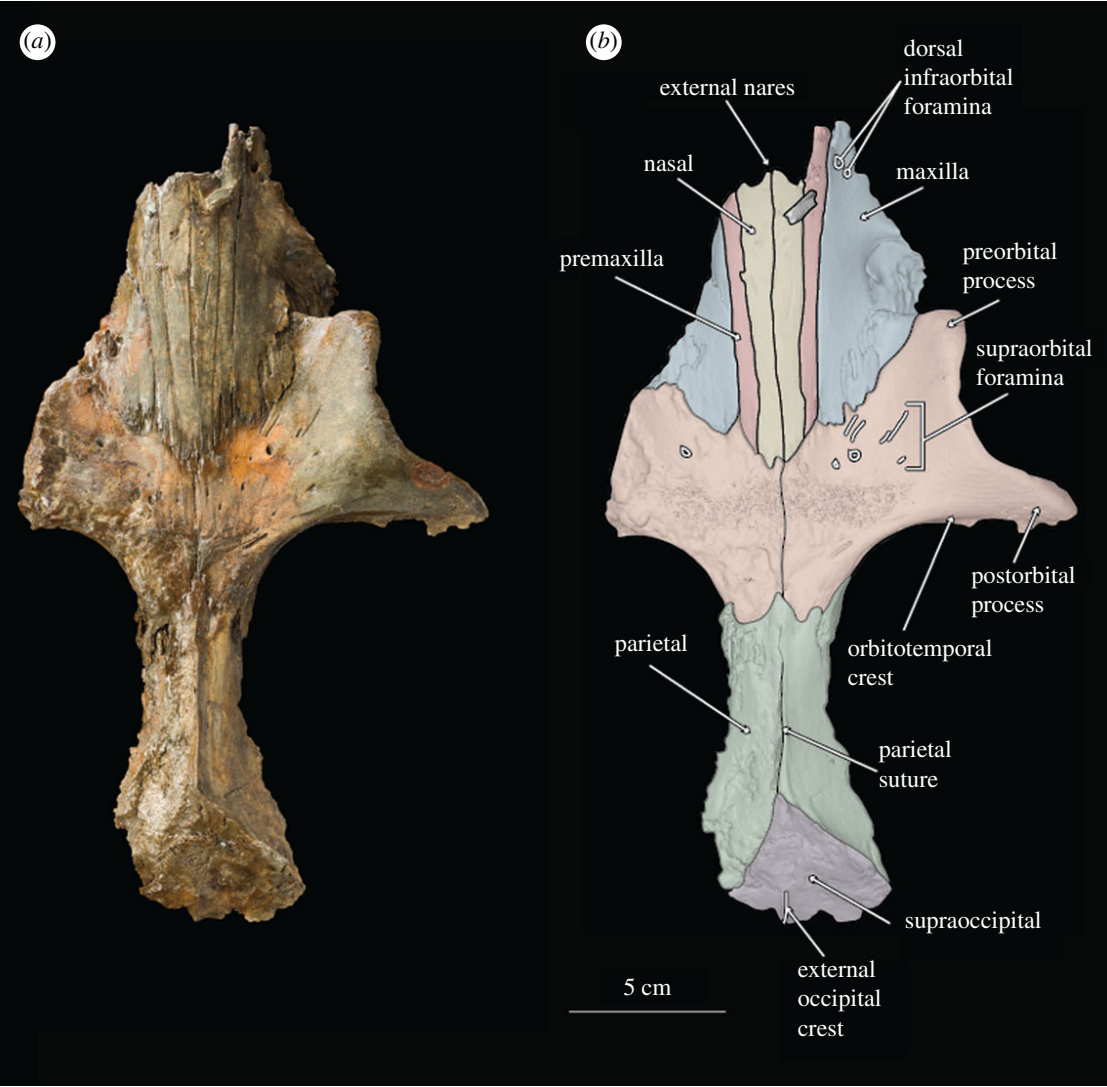
(*a*) Photograph of holotype skull of *B. osedax* (USNM 539939) in dorsal view; (*b*) line art superimposed on 3D model of holotype skull with colour to distinguish bones.

**Figure 2.**
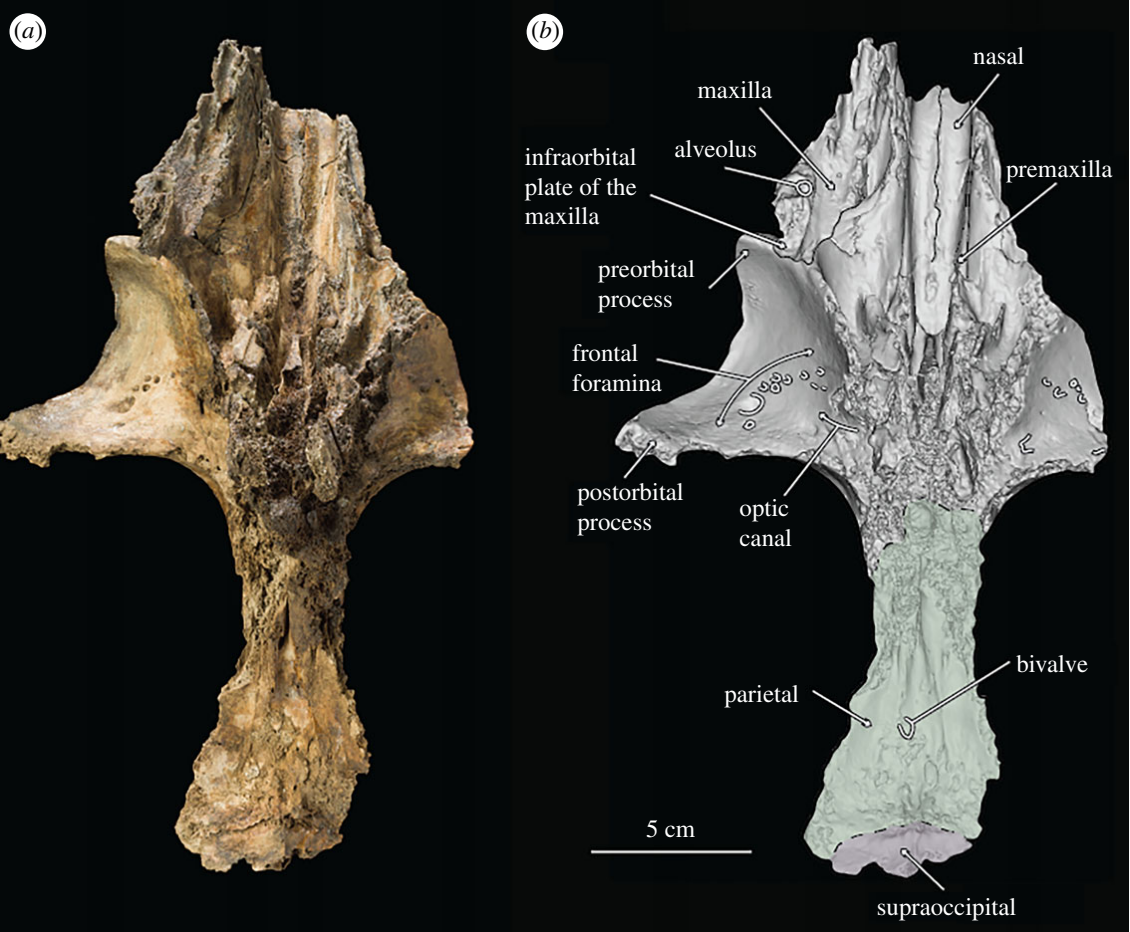
(*a*) Photograph of holotype skull of *B. osedax* (USNM 539939) in ventral view; (*b*) line art superimposed on 3D model of holotype skull.

**Figure 3.**
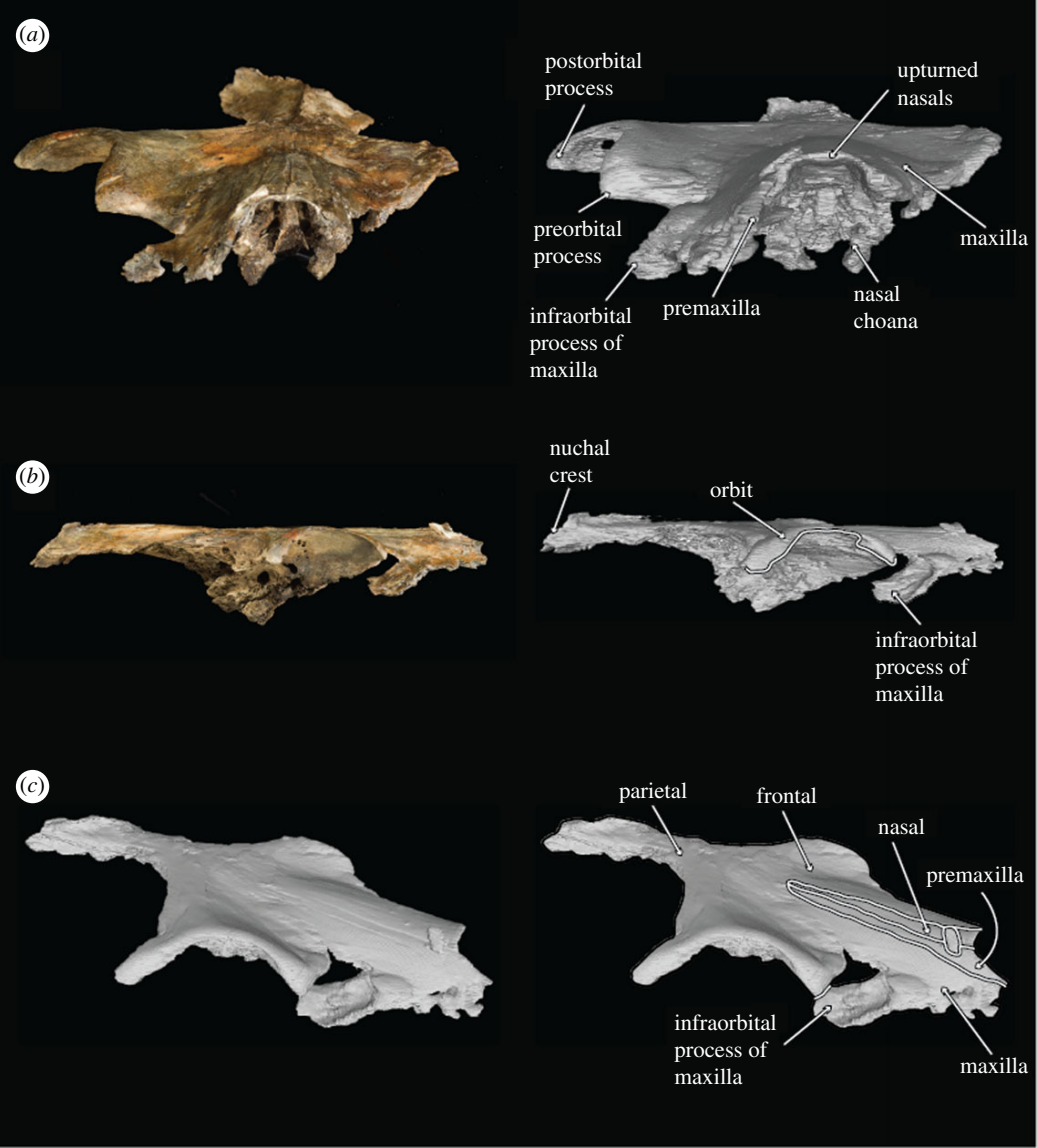
Photographs and 3D models of holotype skull of *B. osedax* (USNM 539939) in anterior (*a*), right lateral (*b*) and oblique (*c*) views with line art superimposed on 3D models.

**Figure 4.**
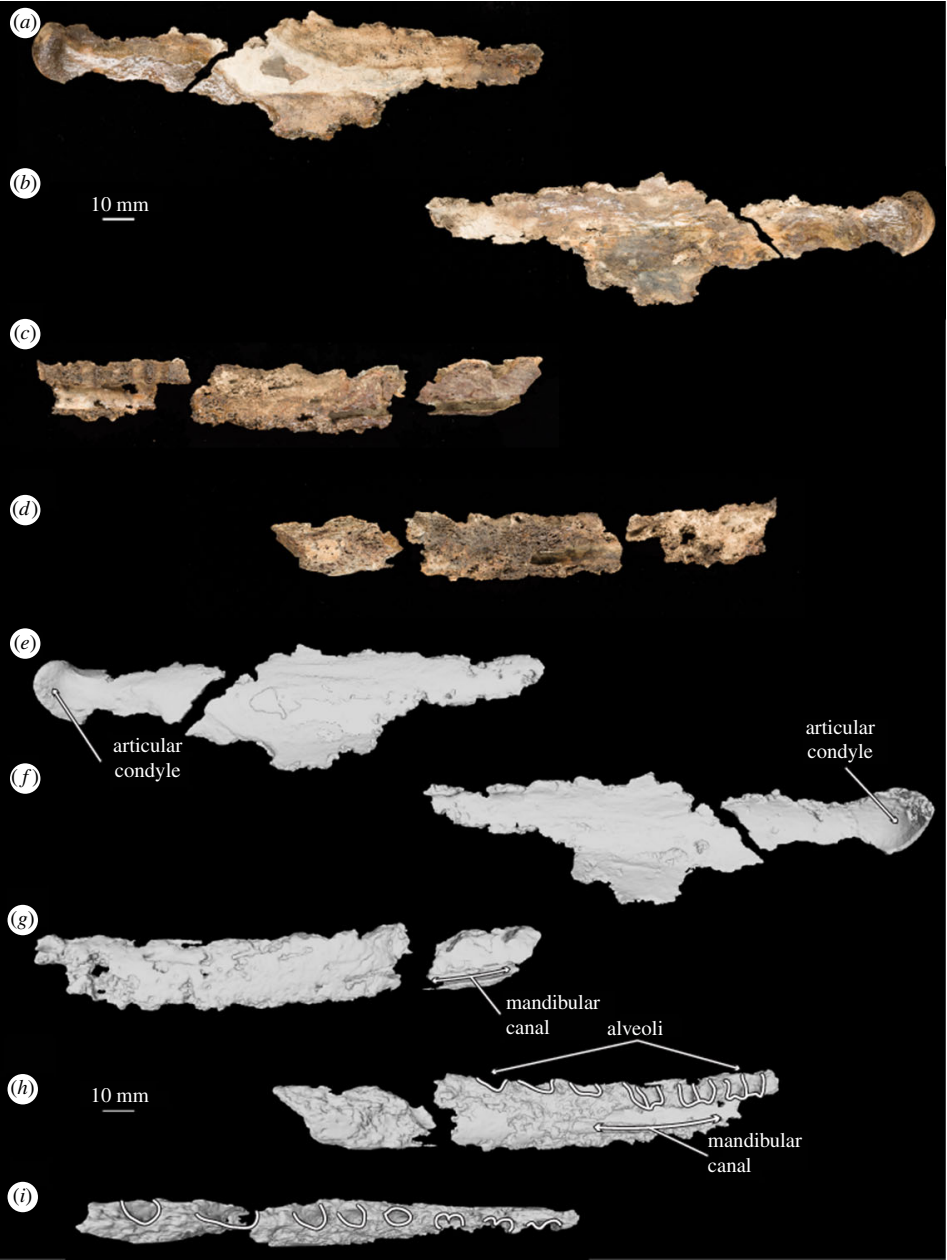
Photograph of holotype right mandible of *B. osedax* (USNM 539939) in lateral (*a,c*) and medial (*b,d*) views. Line art superimposed on 3D model of the holotype mandible in lateral (*e,g*), medial (*f,h*) and dorsal (*i*) views.

**Figure 5.**
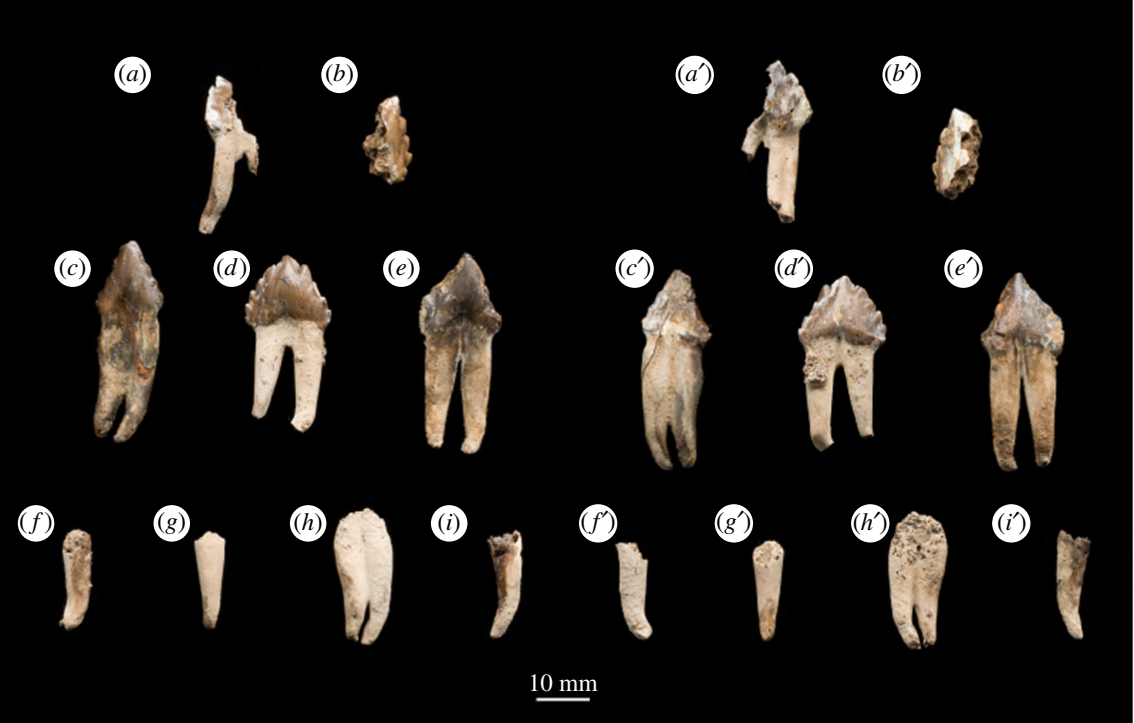
Photograph of holotype teeth of *B. osedax* (USNM 539939) in labial (*a–i*) and lingual (*a‘–i’*) views. Labels (*a–i*) correspond to tooth assignments in the description.

**Figure 6.**
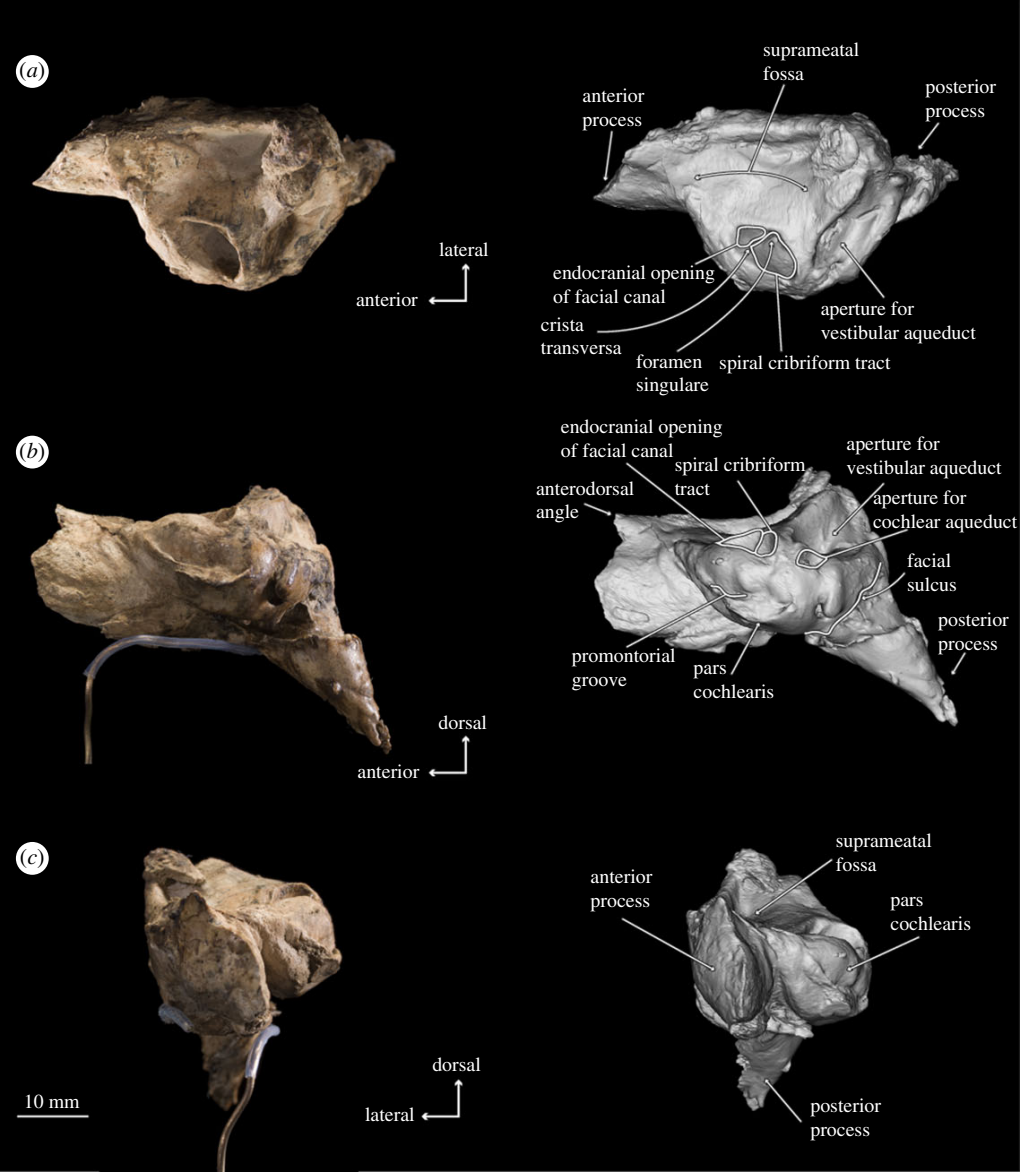
Photographs and 3D models of holotype right periotic of *B. osedax* (USNM 539939) in dorsal (*a*), medial (*b*) and anterior (*c*) views with line art superimposed on 3D models.

**Figure 7.**
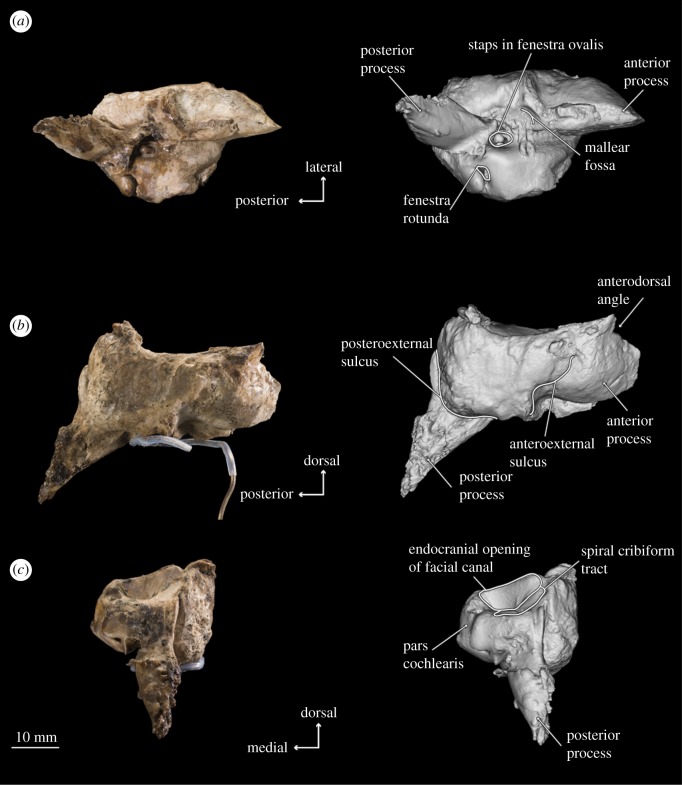
Photographs and 3D models of holotype right periotic of *B. osedax* (USNM 539939) in ventral (*a*), lateral (*b*) and posterior (*c*) views with line art superimposed on 3D models.

**Figure 8.**
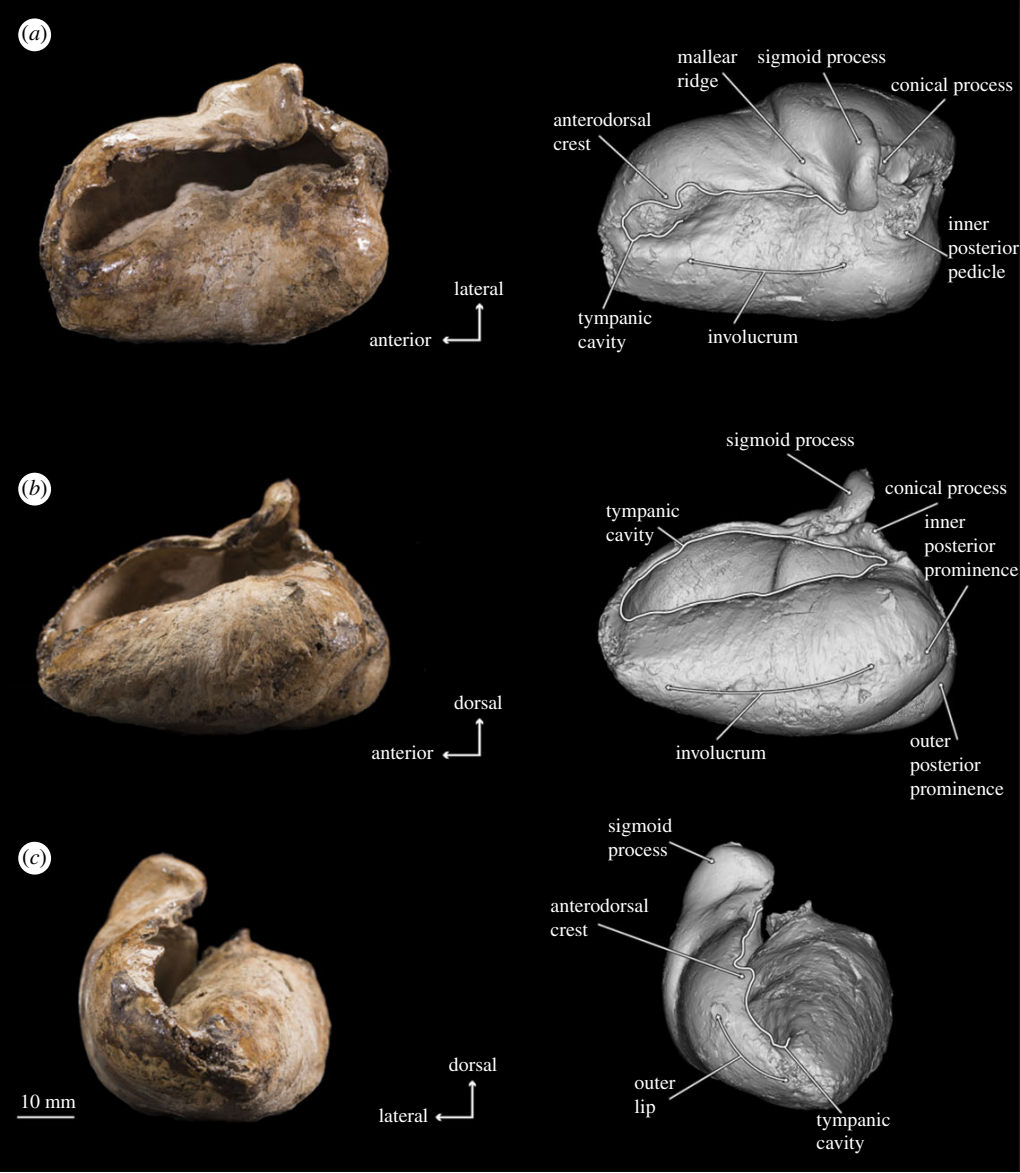
Photographs and 3D models of holotype right tympanic bulla of *B. osedax* (USNM 539939) in dorsal (*a*), medial (*b*) and anterior (*c*) views with line art superimposed on 3D models.

**Figure 9.**
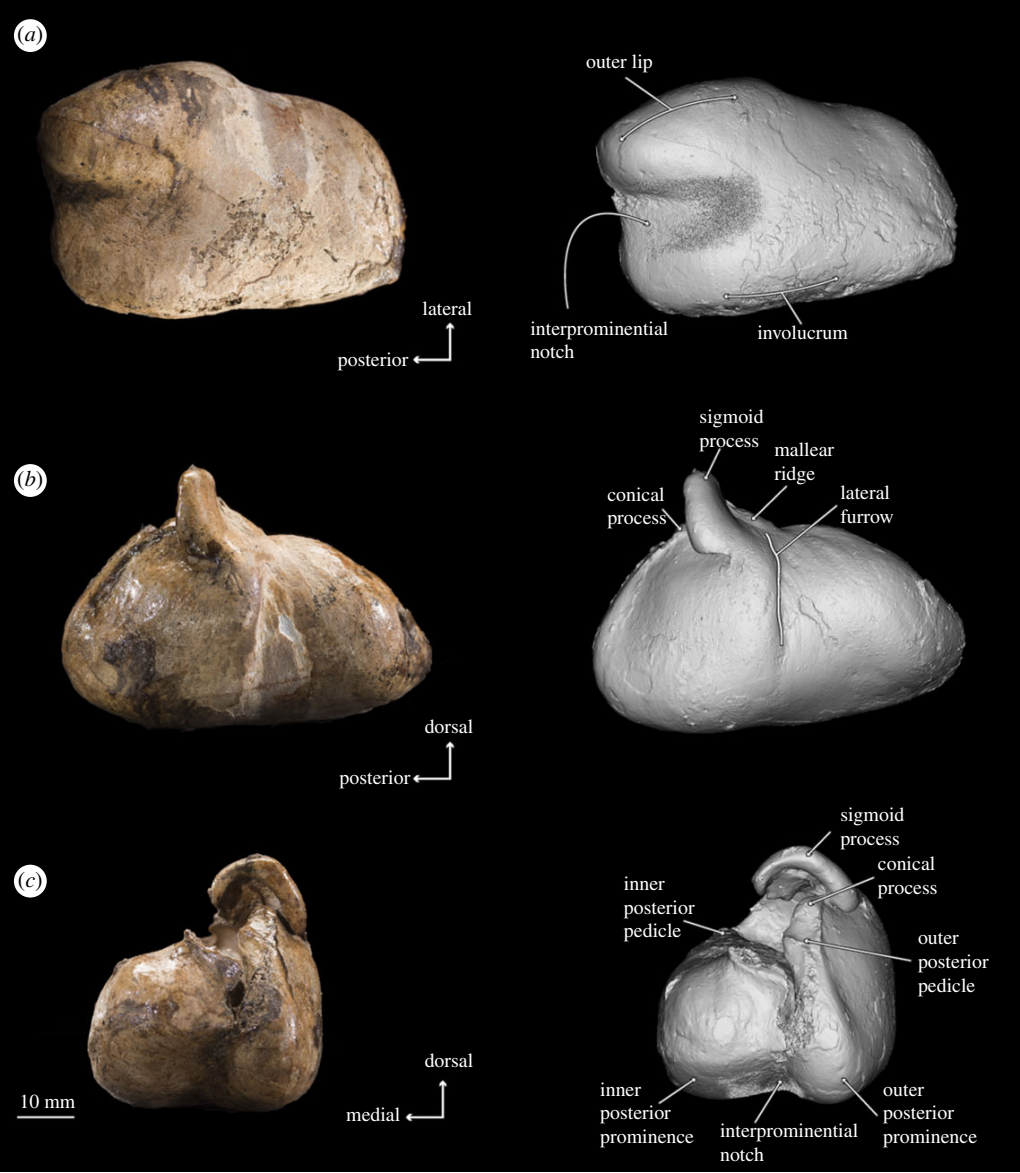
Photographs and 3D models of holotype right tympanic bulla of *B. osedax* (USNM 539939) in ventral (*a*), lateral (*b*) and posterior (*c*) views with line art superimposed on 3D models.

**Figure 10.**
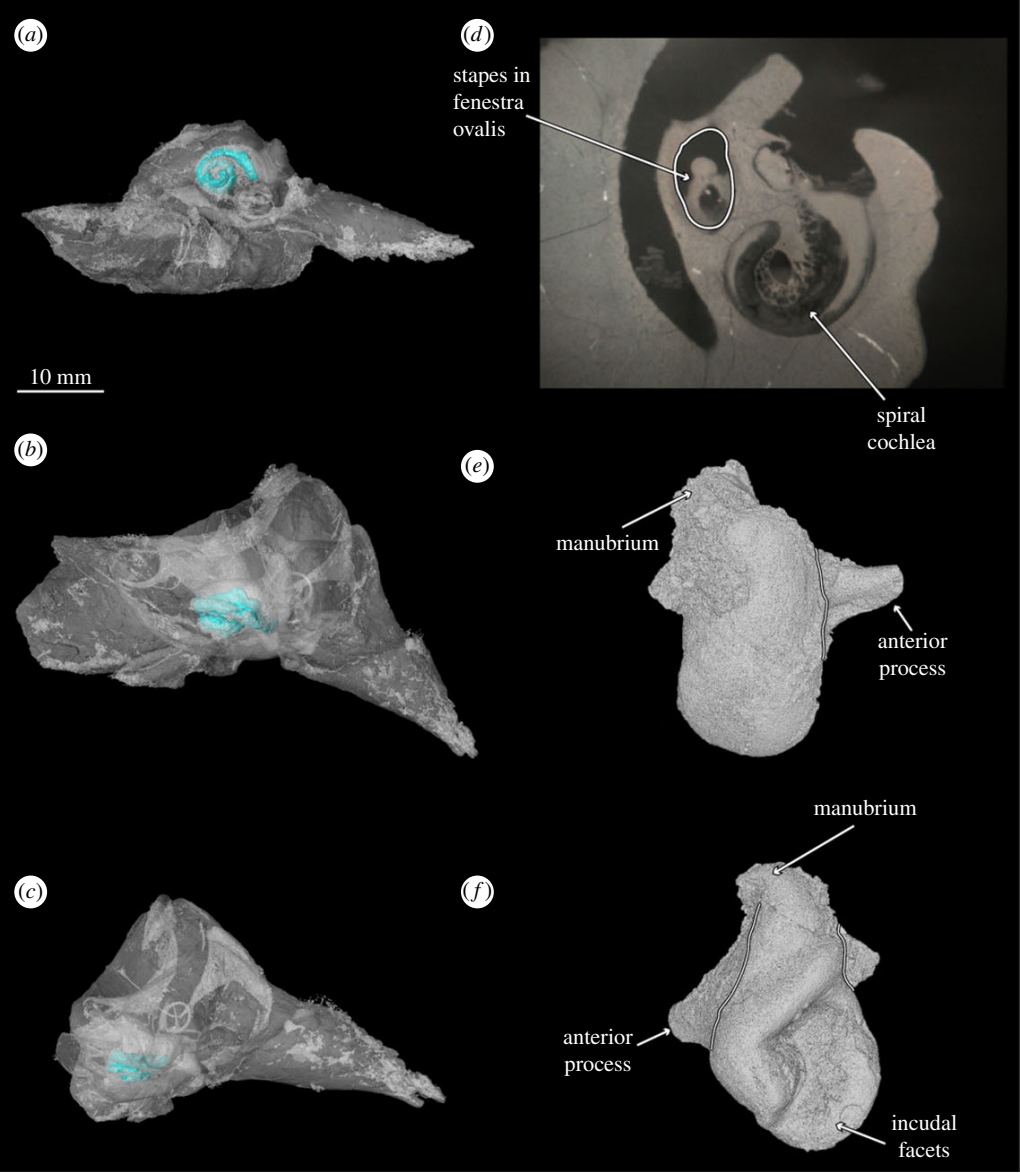
Three-dimensional models of holotype right periotic of *B. osedax* (USNM 539939) in ventral (*a*), medial (*b*) and anterior (*c*) views. The periotic is partially transparent to showcase the spiral cochlea (blue) *in situ* within the pars cochlearis. (*d*) Cross-sectional CT scan of the pars cochlearis revealing the spiral cochlea and the stapes *in situ* in the fenestra ovalis. (*e,f*) Three-dimensional models of the holotype right incus of *B. osedax* (USNM 539939).

**LSID.** urn:lsid:zoobank.org:pub:EDB8544C-F109-453D-BFD5-8A41CFEDE90C

**Etymology.** The specific epithet draws inspiration from *Osedax*, a genus of bone-eating annelid that bores into cetacean skeletons on the deep seafloor. The holotype specimen of *Borealodon* preserves abundant boreholes from this annelid.

**Holotype.** USNM 539939, consisting of a partial skull including a vertex, right periotic and tympanic bulla, and portions of right mandible with dentition.

**Type Locality.** USNM 539939 was discovered and collected by James L. Goedert and Gail H. Goedert along the southern shore of the Strait of Juan de Fuca, just west of Murdock Creek, Clallam County, WA, USA. This locality is the equivalent of LACM locality 5123 and is the type locality of *Behemotops proteus* [14]. Precise coordinates are 48°09'24.0″ N, 123°52'11.3″ W. Approximately 0.8 m east from the LACM locality 5412, which is the type locality of *Olympicetus avitus* [13]. LACM locality 5123 is Paleobiology Database locality number 58964.

**Formation.** Pysht Formation.

**Age.** Rupelian. Murdock Creek is loosely considered the boundary between the Pysht Formation to the west and the Makah Formation to the east, thus placing the type locality of *Borealodon* in the lower Pysht Formation, as summarized by Vélez-Juarbe [13]. Microfossil assemblages indicate an age spanning from the Rupelian to Aquitanian [18–20]. The type locality of *Borealodon* is roughly between the first and third localities sampled by Prothero *et al.* [21] for magnetostratigraphy. These authors correlated the locality with Chrons C11r-C10r, suggesting an age of 30.6–28.3 Ma (Rupelian). This is consistent with the interpretation of previous fossils in the area [13].

**Diagnosis.** *Borealodon* is identified as a Neoceti based on the following derived characters, absent in stem cetaceans: loss of sagittal crest; supraoccipital shield anterodorsally inclined. *Borealodon* can be identified as belonging to the lineage of Mysticeti based on the following derived characters: presence of a maxillary infraorbital plate; and a triangular supraoccipital shield. *Borealodon* is differentiated from Mammalodontidae by having a posterior portion of the maxilla situated lateral to the nasals and the premaxilla (character 20, state 2); a deep suprameatal fossa of the periotic (character 218, state 0); and a medial lobe of the tympanic bulla that is transversely wider than the lateral lobe (character 244, state 0). *Borealodon* is also differentiated from Aetiocetidae in having an anterior process of the periotic that is dorsoventrally deepest within the anterior 50% of the anterior process (character 180, state 1); a periotic with a proximal opening of the facial canal that is larger than the aperture for the vestibular aqueduct (character 190, state 0); and a medial lobe of the tympanic bulla that is transversely wider than the lateral lobe in ventral view (character 244, state 0). *Borealodon* preserves the following combination of character states: a frontal that lies at the same dorsoventral height as the nasals (character 86, state 1); and a periotic with a crista transversa that is deeply recessed within the internal acoustic meatus (character 219, state 1).

## 4. Description

Anatomical terminology follows Mead & Fordyce [22]. A description of the skull refers to either the right or left side, whichever is most complete, unless otherwise stated. Any asymmetry observed is noted.

### 4.1. Skull

USNM 539939 preserves a fragmented but undistorted skull, including a partial cranium. Elements of the posterior ends of the nasals, premaxillae, maxillae, frontals, parietals and the supraoccipital are preserved. USNM 539939 preserves a complete right tympanoperiotic complex in isolation from the holotype skull, as well as fragments of a mandible and elements of as few as seven and as many as nine teeth. As preserved, the skull is 261 mm in length; we estimate *Borealodon* to have measured approximately 1.9 m in total length based on substituting the width of the postorbital processes of the frontal (measured from the midline to the right postorbital process and doubled) for bizygomatic width, which is surely an underestimate based on comparison to other stem mysticetes such as *Janjucetus* and *Aetiocetus* [23].

### 4.2. Premaxilla

USNM 539939 preserves the ascending process of the premaxilla on both sides—about 100 mm on the right and 84 mm on the left. The lateral and medial margins of the ascending process are parallel overall, giving it a rectangular shape overall. The exception is near their anterior termination, where the lateral margin continues straight anteriorly, while the medial margin deflects laterally to accommodate the external nares. Posteriorly, the ascending process forms an interdigitating suture with the frontal that is firm, but not fully fused. This suture is anterolaterally inclined at 45° from the midline. Laterally, the ascending process of the premaxilla is in firm contact with the ascending process of the maxilla, but the suture is ankylosed only near the posterior margin of the suture. Medially, the ascending process of the premaxilla firmly contacts but is not fused to the nasal. The ascending process of the premaxilla reaches posteriorly approximately to the anteroposterior midpoint of the orbit. The left premaxilla extends further posteriorly than the right, though it is unclear if this represents true asymmetry or is a result of taphonomic processes.

In anterior or lateral view, the ascending process of the premaxilla is relatively in the same plane as the nasal. Posteriorly, it lies about 3 mm ventral to the nasals and exhibits no ventral sloping. The result of this is that all the premaxilla, maxilla and nasal contact the frontal at nearly the same dorsoventral elevation. Anterior to the posterior margin of the nares, however, the premaxilla descends sharply ventrally, suggesting that the rostrum was in a plane well below the nasals.

### 4.3. Maxilla

The maxilla of USNM 539939 is damaged laterally but preserved medially where it contacts the premaxilla but not the nasals, and posteriorly where it contacts the frontals. The rostral portion preserves two dorsal infraorbital foramina just anterior to the anterior margin of the nasals. The larger of the two is approximately 5 mm in diameter and the smaller is approximately 1 mm in diameter. Medial to the infraorbital foramina is the maxillary–premaxillary suture, which as described above, is tight but unfused.

Posteriorly, the ascending process of the maxilla contacts the frontal as a continuation of the 45° suture made by the frontal and the premaxilla. This suture extends anteriorly until near the antorbital notch, where the frontal–maxillary suture turns laterally to demarcate the preorbital process of the frontal. At this lateral margin, the maxilla preserves a badly damaged, dorsoventrally thin infraorbital plate. The infraorbital plate preserves an alveolus and a second indentation that probably represents the alveolus for a second root. We infer that these are the alveoli for the posteriormost upper tooth as they are approximately 1 cm anterior to the level of the anterior border of the preorbital process of the frontal.

In lateral view, the maxilla slopes anteroventrally away from the frontal. This results in an ascending process of the maxilla that is well elevated and distinct from what little of the rostral portion of the maxilla is preserved. Anteriorly, the ascending process also slopes ventrolaterally away from the premaxilla, such that the lateral margin of the ascending process lies well below the medial margin. Laterally, a small section of the infraorbital plate extends posteriorly; though minimal, this section is transversely broad and dorsoventrally thin.

### 4.4. Nasal

Anteriorly, the nasal has a slight U-shaped margin, with the lateral and medial margins extending slightly farther anteriorly than the rest. The result is an external nares that is slightly bifurcated at the midline by the two nasals. The medial and lateral margins of the nasal diverge slightly anteriorly such that overall the nasal is transversely widest anteriorly and tapers to a thinner transverse width posteriorly. Posteriorly, the medial and lateral margins converge slightly such that the posteriormost element of the nasal is at its midline. This results in a W-shaped suture between the frontals and nasals at the vertex. The nasal is tightly sutured to the frontal at its posterior margin; the suture is interdigitating and somewhat damaged, obscuring its exact shape. Posteriorly, the nasals extend onto the frontal posteriorly about 47 mm from the level of the antorbital notch. The suture between the nasals and premaxilla is tight but unfused along its entire length. In lateral view, the nasals deflect dorsally at their anterior margin as they reach the external nares.

### 4.5. Frontal

At their posterior margin, the frontals meet the parietals in a V-shaped suture. Though the parietal preserves a faint sagittal crest, it terminates just before this suture and does not extend onto the frontal. The suture between the left and right frontal is visible for its entire length, extending anteriorly from the parietal to the posterior margin of the nasals.

The left frontal is poorly preserved overall—only minimal elements of the supraorbital process are preserved and neither the preorbital nor postorbital processes are present. Faint foramina are visible near what is preserved of its anterior margin where the antorbital process and lacrimal would have been.

The right frontal, however, is nearly complete, and lacks only the posterior margin of the postorbital process. The orbitotemporal crest extends from the anterior margin of the parietal to about 25 mm from the distal termination of the postorbital process. It is coincident with the posterior border of the supraorbital process of the frontal as in stem cetaceans, llanocetids, *Coronodon* and mammalodontids; it does not extend onto the dorsal surface of the supraorbital process as in aetiocetids and chaeomysticetes. The orbit measures 73 mm from the distal terminations of the preorbital and postorbital processes of the frontal. The postorbital process extends laterally beyond the preorbital process, such that overall the orbit points anterolaterally. Within the orbit, the optic canal exits the braincase with a strong anterior orientation.

The dorsal surface of the right frontal is marked by several foramina of varying depths and shape; some appear as deep ovoid holes with little to no sulci, while others are shallow, and bear extended sulci. A particularly deep foramen can be seen just posterior to the ascending process of the maxilla, and measures 3 mm in diameter. The ventral surface of the frontal also shows deep foramina, with a cluster of at least eight deep foramina opening into the orbit. Medially, these are angled ventrally, but moving laterally, these foramina take on a more lateral inclination.

### 4.6. Parietal

The parietals are preserved only at the midline; the right parietal is the better preserved of the two. None of the temporal wall remains. However, at this apex, the parietals are transversely thin and notably constricted as in archaeocetes, toothed mysticetes such as *Mammalodon* and *Coronodon*, and some eomysticetids. The parietal bears a faint sagittal crest, approximately 10 mm at its highest point, that terminates about 10 mm posterior to the suture with the frontal.

The parietal measures about 89 mm in length at the midline from its contact with the frontal to its contact with the supraoccipital. The right parietal contacts the supraoccipital at the midline and continues posterolaterally from the midline where it underlies the nuchal crest. Here, the parietal descends posterolaterally, so that it lies fully below the nuchal crest.

### 4.7. Supraoccipital

Only the anteriormost margin of the supraoccipital is preserved. Here, the supraoccipital forms a triangular apex where it contacts the parietals. Posteriorly, what is preserved is only slightly sloped anteroposteriorly. The 44 mm of the nuchal crest visible on this specimen overhang the parietal and are relatively straight, not curved, giving the occipital shield a triangular shape without rounded margins. At the posteriormost region of the supraoccipital shield is a small ridge, approximately 10 mm high, that probably represents the anterior portion of an external occipital crest. On either side of the external occipital crest lies a deep fossa along the surface of the supraoccipital.

### 4.8. Periotic

In ventral view, the anterior process appears anteriorly pointed and triangular, with a flat medial margin and gently convex lateral margin. The anterior process is sharply keeled at its anterior termination. The pars cochlearis is bulbous with a rounded medial margin, and is longer anteroposteriorly than it is transversely broad. The posterior process deflects laterally from the pars cochlearis and anterior process, with an overall elongate shape that narrows to a blunt tip at its posterior margin. Within the posterior half of the pars cochlearis is the fenestra ovalis, which appears rounded and relatively large compared to that of aetiocetids such as *Chonecetus* and opens anteriorly. The stapes (see §4.10) is *in situ* in the fenestra ovalis. Anterolateral to the fenestra ovalis is the mallear fossa, which is shallow and longer anteroposteriorly than transversely wide. The lateral tuberosity lies lateral to the mallear fossa and appears as a blunt ridge with a slight anterior deflection. Medial to the fenestra ovalis and on the posterior surface of the pars cochlearis is the fenestra rotunda, a crescent-shaped hole in ventral view that opens posteromedially. Slightly overlying this is a triangular but relatively dull protrusion pointing almost exactly ventrally from the pars cochlearis.

In dorsal view, the suprameatal fossa appears large, elongated anteroposteriorly, widened transversely and dorsoventrally deep such that it comprises most of the dorsal surface of the periotic. The internal acoustic meatus is elongated into an oval, but is elongated anterolaterally rather than anteriorly. The endocranial opening for the facial canal is teardrop-shaped, with the tail pointed anteriorly. The opening for the facial canal is separated from the spiral cribriform tract by the crista transversa, which is deeply recessed within the internal acoustic meatus. The spiral cribriform tract is then separated from the foramen singulare by a slender, narrow crest that is recessed even further into the internal acoustic meatus. Posterior to the internal acoustic meatus is a deep basin containing the apertures for the vestibular and cochlear aqueducts. The elliptical aperture of the vestibular aqueduct is dorsolateral to and significantly larger than the aperture for the cochlear aqueduct, which is circular and small. In this dorsal view, the two apertures are nearly aligned transversely, but in medial view, the aperture for the cochlear aqueduct appears just posterior to the aperture for the vestibular aqueduct.

In lateral view, there are deep posteroexternal and anteroexternal sulci, which together frame a rectangular region of the lateral face of the periotic. At the ventral margin at about the midpoint of the periotic is the lateral tuberosity, which appears in lateral view as a thin protrusion with a dull, ventrally oriented point. The anterodorsal angle extends dorsally beyond the posterodorsal angle, and is overall more slender and pointed than the comparatively rounded and blunt posterodorsal angle.

In medial view, the apertures for the vestibular and cochlear aqueducts open posterodorsally; the aperture for the vestibular aqueduct is mostly visible but partially obscured by the swelling of the pars cochlearis. The anterior process in medial view is elongated anteroposteriorly with two distinct projections: one at the dorsoventral midpoint and one at the dorsal tip, which represents the anterodorsal angle. The anterodorsal angle is a sharp, spear-shaped projection. The more ventral projection is rounded and more blunt than the anterodorsal angle. The pars cochlearis retains its bulbous shape in medial view, but the promontorial groove is more visible than it was in ventral view. The endocranial opening of the facial canal is also visible in this view, with the crista transversa clearly separating it from the spiral cribriform tract. On the posterior edge of the pars cochlearis, the fenestra rotunda is as visible as it was in ventral view; the triangular protrusion on its posterior margin that overhangs the fenestra rotunda is more visible in medial view. The posterior process appears cone-shaped in medial view. The facial sulcus is clearly visible as a deep, wide canal leading from the distal opening of the facial canal and over the posterior process dorsally.

### 4.9. Tympanic bulla

In ventral view, the bulla is smooth with minimal damage or protrusions on its surface. The outer posterior prominence extends further posteriorly than the inner posterior prominence and has a more pointed shape. Between the two prominences, the interprominential notch is transversely broad. The anterior termination of the bulla is rounded and anteriorly convex.

In dorsal view, the tympanic cavity is transversely thin and obscured by the outer lip. The medial margin of this cavity is smooth as it gently rises to the involucrum surface, which is rounded throughout its length. The involucrum is transversely inflated and dorsally convex. At about the anteroposterior midpoint of the bulla is a transverse ridge within the tympanic cavity. On the lateral side, the mallear ridge is a slender, narrow structure pointing anterolaterally at about 45°. Similarly, the sigmoid process also points anterolaterally but at a shallower angle. The sigmoid process is transversely flat anteriorly but distinctly convex posteriorly. In dorsal view, it extends to overhang the tympanic cavity and is notably robust compared to the slender sigmoid processes of other stem mysticetes, such as *Salishicetus* [15]. Finally, the inner and outer posterior pedicles lie posterior to the sigmoid process and are aligned transversely.

In medial view, the anterior margin of the tympanic bulla tapers to a blunt apex. The dull, nearly flat involucral ridge extends from this apex to the posterior margin of the bulla. Dorsal to the posterior margin of this ridge are the inner and outer posterior pedicles: the inner posterior pedicle is clearly visible in medial view and distinctly pointed, while the outer posterior pedicle is partially obscured by the inner and lies flat, ventrally below the level of the inner. Slightly anterior to the posterior pedicles, the conical process is dorsoventrally thickened and rounded in medial view, creating a notably inflated shape when compared with other stem mysticetes. The sigmoid process rises dorsally from the outer lip with a slight posterior angle so that it overhangs the conical process. Anteriorly, the sigmoid process slopes steeply into the blunt mallear ridge, which is barely visible in medial view as it leads into the shallow but V-shaped groove of the sulcus for chorda tympani.

When viewed laterally, the sigmoid process rises dorsally from the outer lip. At its base is the sigmoid fissure, a relatively deep groove that forms a nearly 90° angle between its ventral and posterior margins. Anterior to this is the lateral furrow, which is bounded posteriorly by a ridge at the anteroposterior midpoint and expanding ventrally into a triangular valley. Finally, the posterodorsal margin of the lateral lobe forms a sharp keel that extends from the outer posterior pedicle to the posterior margin of the bulla.

### 4.10. Auditory ossicles

USNM 539939 preserves an isolated right malleus. The articular surface where the malleus meets the incus preserves a raised ridge and a recessed, flat surface. The anterior process is elongated and extended away from the manubrium and terminates in a rounded, blunt point. The manubrium is transversely broad, rounded and short relative to the manubrium of *Llanocetus*.

The stapes of USNM 539939 is preserved *in situ* within the fenestra ovalis of the periotic. The structure is elongate with a narrow centre and an expanded distal head that is rounded where the stapes would meet the incus.

### 4.11. Mandible and dentition

USNM 539939 preserves elements of as few as seven and as many as nine teeth with both roots and crowns, as well as incomplete tooth roots. All of the crown-bearing teeth show thin, raised striations down their length on the lingual surface extended upward from the base of the crown towards but not on the apex. Based on comparison with other toothed mysticetes such as *Salishicetus, Aetiocetus*, *Fucaia* and *Janjucetus*, we conservatively interpret six right lower postcanine teeth and three indeterminate teeth. The morphology of the accessory cusps and the curvature of the tooth roots support this interpretation, as does the presence of right mandibular elements but few rostral elements. However, the possibility that the six identified teeth instead represent left upper postcanine teeth cannot be ruled out. All of the teeth preserving crowns preserve an enamel shelf on the lingual surface, identified here as the entocingulum. There is no evidence of an ectocingulum in any tooth.

Tooth A (figure 5*a*) was figured by Kiel *et al.* [12] (figure 2*e*). This tooth preserves half of the crown, a small portion of one root and a nearly complete second root. The crown preserves the apical cusp, which shows minimal apical wear, and four accessory cusps with heavy occlusal wear. This occlusal wear results in the accessory cusps being blunt and poorly differentiated from one another, reminiscent of those seen in the more heavily worn teeth of *S. meadi*. The roots are fully separated and bear a strong lingual and slight posterior curvature. As noted by Kiel *et al.* [12], the root of tooth A preserves at least three distinct *Osedax* boreholes.

Tooth B (figure 5*b*) represents a single half of the tooth crown. It preserves the apical cusp with no apical wear, and either four or five accessory cusps with only minimal occlusal wear. The breakthrough of the crown reveals that the enamel layer appears thin.

Tooth C (figure 5*c*) preserves a crown and both roots which curve labially. The roots are nearly completely fused and probably were preserved in a single alveolus lacking an isthmus. In this, tooth C resembles the possible first premolar of *S. meadi*. The roots narrow transversely as they near the crown such that the crown is actually transversely wider than the root beneath it. The apical cusp is preserved and worn to a blunt tip, and the accessory cusps preserve heavy occlusal wear such that the number of accessory cusps is uncertain. Tooth C does not preserve any obvious *Osedax* boreholes.

Tooth D (figure 5*d*) is the most complete and best-preserved tooth of *B. osedax*. It was figured by Kiel *et al.* [12] (figure 2*c*) and used in the complexity analysis by Peredo *et al.* [6]. The tooth is double rooted, with the proximal ends of the roots partially broken since they were originally figured by Kiel *et al.* [12], and the roots have a slight labial and posterior curvature. The two roots are separate for most of their length but fuse just ventral to the crown, though they remain distinguished by a deep groove that continues into the crown and gives it a slight heart-shape in labial view. The crown preserves a complete apical cusp, four anterior accessory cusps and five posterior accessory cusps, all of which demonstrate minimal occlusal wear. The accessory cusps are overall triangular and the divisions between the cusps are shallow relative to other toothed mysticetes such as *Coronodon* and *Salishicetus*. As noted by Kiel *et al.* [12], tooth D preserves several *Osedax* boreholes throughout its roots.

Tooth E (figure 5*e*) preserves most of the crown and both roots. As with tooth D, the roots are separate for most of their length and fuse only near the crown. They also exhibit a slight labial and posterior curvature at their proximal terminations. As with tooth C, the accessory cusps show occlusal wear, making it difficult to distinguish the number of anterior accessory cusps, though we interpret four posterior accessory cusps. Though not figured by Kiel *et al.* [12], tooth E does preserve several boreholes.

Teeth F–I (figure 5*f–i*) are tooth roots only with no crowns preserved. Of these, only tooth H preserves a double tooth root, but it is unclear if teeth F, G and I are single-rooted teeth or simply single crowns of double-rooted teeth. All four teeth preserve a slight posterior and labial curvature at their proximal terminations. Tooth H was figured by Kiel *et al.* [12] (figure 2*d*) and preserves several boreholes throughout both roots. The other three teeth do not preserve any obvious boreholes.

The right dentary of USNM 539939 is in several pieces, including one larger posterior section and one smaller anterior section, with the latter so poorly preserved that pieces of it crumble readily. Posteriorly, the mandible preserves an articular condyle that is round and kobold-like with a deeply pitted and rugose surface. In posterior view, the articular condyle is keeled dorsally and broad transversely. Anterior to the articular condyle, the body of the mandible thins quickly so that the condyle appears to be connected by a thin neck to what probably was the rest of the mandible corpus. Anterior to this level, the lateral surface of the mandible is preserved but is badly damaged, so that any morphology is obscured.

A second anterior piece preserves approximately 156 mm of the alveolar surface. This mandibular element preserves alveoli for at least 12 tooth roots. Based on size, orientation and spacing, we interpret these alveoli to correspond to three single-rooted teeth and three double-rooted teeth. Deep breaks in the body of the mandible expose the internal morphology, revealing that the mandibular canal is low in the body of the mandible and the tooth roots penetrate to the level of the mandibular canal, as in most toothed cetaceans [24].

## 5. Discussion

### 5.1. Phylogenetic position and taxonomic identity

The skull of *Borealodon* is poorly preserved, making phylogenetic coding difficult. In general, the skeletal material for genotypic species of Aetiocetidae are largely non-overlapping: many named taxa lack isolated tympanoperiotics, complete crania, dentition or mandibles. For example, there are no described periotics for *Aetiocetus* spp.; equally, there is no cranial material aside from the elements directly surrounding the tympanic cavity in *Salishicetus*. Testing the position of *Borealodon* relative to mammalodontids is particularly difficult because many of the mammalodontid synapomorphies relate either to the rostrum or to the bizygomatic width, neither of which is preserved nor can be estimated.

Our analysis does not include recently described stem mysticetes such as *Mystacodon*, *Llanocetus*, *Coronodon* and *Niparajacetus*, which have not been observed by the authors. However, respective phylogenetic analyses recover these taxa basal to Mammalodontidae, and are therefore unlikely to affect the phylogenetic position of *Borealodon*. Moreover, the size, spacing and overall dental morphology of all of these aforementioned taxa do not correspond to *Borealodon*.

The most recent analysis of mysticete phylogeny using this matrix [15] recovered Mammalodontidae (*Mammalodon* + *Janjucetus*) as a monophyletic clade based on the following 10 synapomorphies: (i) the premaxilla exposed in the palate only anterior to the maxilla (character 11, state 1); (ii) a short rostrum that is less than 40% of the condylobasal length (character 15, state 1); (iii) a posterior portion of the maxilla situated lateral to the nasal (character 20, state 1); (iv) a wide antorbital notch measuring between 40 and 54% of the bizygomatic width (character 42, state 0); (v) an anterior edge of the narial fossa located in the anterior quarter of the rostrum (character 47, state 1); (vi) a broad rostrum overall measuring over 80% of the rostrum length (character 49; state 1); (vii) short nasals measuring less than 40% of the bizygomatic width (character 59, state 1); (viii) a preorbital process of the frontal that is rounded off with an anteriorly convex margin (character 83, state 1); (ix) an anterior process of the periotic preserving a deep pit on the lateral surface (character 166, state 0); (x) a shallow or absent suprameatal fossa of the periotic (character 218, state 1).

**Figure 11.**
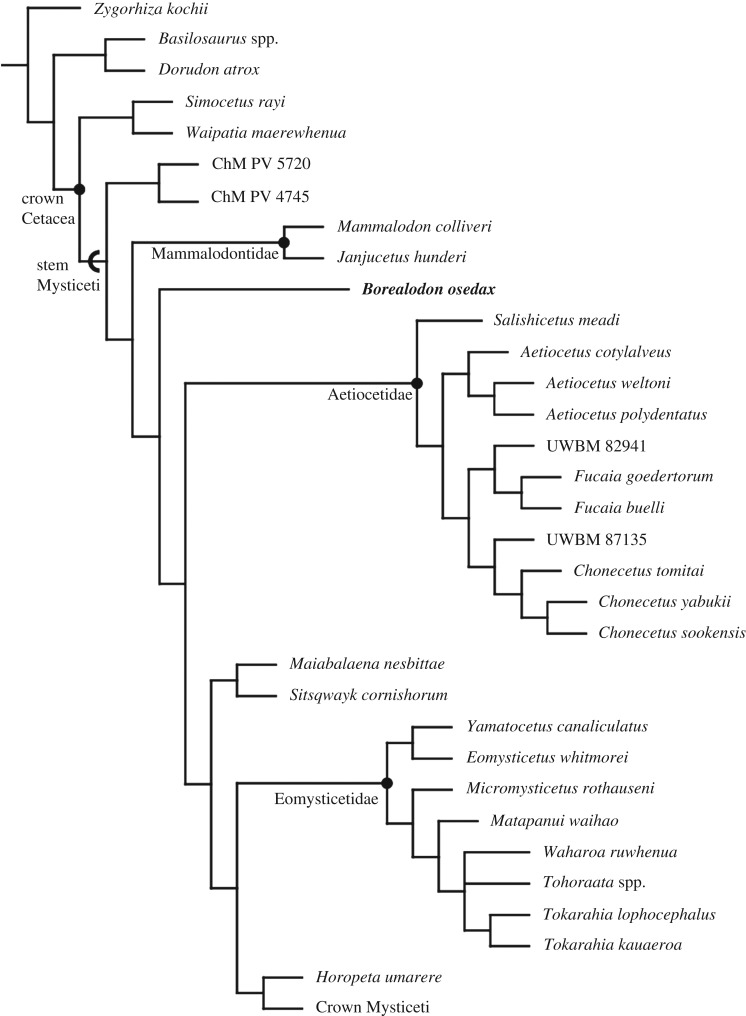
Phylogenetic relationship of *B. osedax* relative to other stem Mysticeti. Cladogram shown is a strict consensus tree. Crown Mysticeti has been collapsed into a single terminal unit for visualization, but relationships therein are unchanged from Peredo & Pyenson [15].

Our phylogenetic analysis recovers similar results; we recover a monophyletic Mammalodontidae based on eight of the aforementioned synapomorphies (characters 11, 15, 20, 42, 47, 49, 59 and 218) and one new synapomorphy: a tympanic bulla with a medial and lateral lobe approximately equivalent in width (character 244) (figure 11). Two previous synapomorphies of Mammalodontidae (characters 83 and 166) no longer inform the monophyly of Mammalodontidae because they are shared with *Borealodon*. *Borealodon* can be coded for three of the nine diagnostic characters of Mammalodontidae recovered by this analysis; all three (characters 20, 218 and 244) exclude *Borealodon* from Mammalodontidae (see diagnosis).

The other well-known clade of toothed, stem mysticetes from the Pacific Northwest region are the aetiocetids. Peredo & Pyenson [15] identified seven synapomorphies of Aetiocetidae. Our analysis recovers a monophyletic Aetiocetidae based on four synapomorphies: characters 173, 180, 190 and 244. Of these, *Borealodon* can be coded for three (characters 180, 190 and 244), and all three exclude *Borealodon* from Aetiocetidae (see diagnosis).

This combination of character states results in *Borealodon* representing a distinct lineage of stem mysticete, crown-ward of Mammalodontidae and stem-ward to Aetiocetidae. This result is recovered in all 359 best score trees (best score of 1595 steps). Thus, despite the challenges in phylogenetic coding of stem mysticetes, there is evidence that *Borealodon* is neither a mammalodontid nor an aetiocetid. Other toothed, stem mysticetes such as *Llanocetus*, *Mystacodon*, *Coronodon* or *Niparajacetus* [25] are a poor fit for its size and observed morphology.

### 5.2. Hearing

We tested the low-frequency limit of hearing following the methods of [26]. The lower limit of hearing frequency in cetaceans can be estimated based on the ratio of the radius of the cochlea at its base with the radius of the cochlea at its apex. Notably, we measured this ratio in *Borealodon* as 6.44; Park *et al.* [26] report an identical value for NMV P173220—identified by the authors as Mammalodontidae indet. The value for *Borealodon* is also comparable with the value of 6.77 reported for *M. colliveri*. Following the methods of [26], we estimate a low-frequency limit of 64.88 Hz; these values are in line with the estimates for NMV P173220 and *M. colliveri* (65.07 Hz and 53.81 Hz, respectively).

Moreover, these values are notably larger than those reported by Park *et al.* [26] for NMV P229119 (7.47 radii ratio, 35.90 Hz low-frequency limit): the only aetiocetid included in their analysis. Inner ear morphology is difficult to reconstruct and is therefore rarely coded in phylogenetic analyses. These results suggest that *Borealodon* may be more similar in its inner ear morphology to mammalodontids than to aetiocetids. Given that *Borealodon* is not a mammalodontid, this may suggest that *Borealodon* occupied a bioacoustic niche similar to mammalodontids, which may represent the plesiomorphic condition shared with mammalodontids and distinct from aetiocetids. Alternatively, if additional fossil material reveals *Borealodon* to instead be an aetiocetid, these results suggest it may have been acoustically convergent with the mammalodontid bioacoustic niche. However, we emphasize that the inner ear morphology has not been described nor measured for any aetiocetid diagnostic to genus level. Accordingly, the bioacoustic diversity within Aetiocetidae remains poorly known.

### 5.3. Feeding

The most complete tooth of *Borealodon* was included in the recent analysis by Peredo *et al.* [6]. These authors found the tooth of *Borealodon* to be less complex (based on orientation patch count as initially developed by Evans *et al.* [27]) than the teeth of aetiocetids such as *A. cotylalveus* and *S. meadi*. This less complex value may be partially driven by a reduction in size of the accessory cusps, though the method is robust to changes in tooth size overall. In this, the teeth of *Borealodon* bear little resemblance to the elaborate, complex teeth of *J. hunderi* or aetiocetids. The cheek teeth of *Janjucetus* have three or four accessory cusps with deep divisions separating each cusp from the next. The teeth of *Borealodon* have as many as five cusps with very shallow divisions between the cusps such that the overall shape of the crown is more continuous. Moreover, the teeth of *Borealodon* lack the extreme curvature of the tooth roots, curved crowns and reduced accessory cusps characteristic of *Aetiocetus* spp. Instead, the overall smaller teeth, with shallow divisions between the accessory cusps and the heavy apical wear, are more reminiscent of *F. buelli*.

Moreover, four of the teeth show occlusal wear on the accessory cusps evocative of, but not as pronounced, as the wear seen in *Salishicetus*. Given the lack of rostral material and the fragmentary nature of the mandible of *Borealodon*, we are cautious in interpreting its feeding mode. However, based on comparable occlusal wear to *Salishicetus* and *Fucaia*, we interpret *Borealodon* as a raptorial feeder that used its teeth for prey processing [15,28].

### 5.4. Taphonomy

First, the cortical surface of nearly every element of USNM 539939 preserves small (less than 0.1 mm) circular boreholes. Previous authors have reported and attributed the boreholes to *Osedax*, a bone-eating annelid that consumes cetacean skeletons on the abyssal seafloor today [11,12]. These boreholes are preserved on the skull, mandibular elements, rib elements and tooth roots. They are not preserved on the ear bones nor on the tooth enamel. Previous authors speculated that the osteosclerotic earbones may have been too hard or lacked sufficient nutrients for *Osedax* to colonize [11]. The lack of boreholes on the tooth enamel supports the former hypothesis; *Osedax* seems to preferentially favour the less dense material—at least in USNM 539939. In particular, teeth A and B (figure 5*a,b*) demonstrate *Osedax* activity in the exposed dentin but not the enamel.

Second, the extent of bioerosion of the underlying cancellous bone, deep to the cortical bone across each element, is so substantial that it has jeopardized the structural integrity of the specimen. This bioerosion is visible along the posterior border of the supraorbital process of the right frontal and the preserved parts of the mandible (figures 1, 2 and 4), which are both adjacent to nearby boreholes on cortical bone. We infer that *Osedax* bioerosion reduced much of the cranial material of USNM 539939 prior to burial.

Third, we note the presence of a single, incomplete bivalve in direct association with ventral surface of the right parietal, along the internal surface of the preserved braincase (figure 2). The bivalve preserves an incomplete external surface that is approximately 2 mm wide and 3 mm long; the overall oval morphology and size closely matches that of other vesicomyid bivalves that are reported in direct association with fossil mysticete skeletal material from fossil whale-falls, such as the one reported by Pyenson & Haasl [29] from the Monterey Formation of California. The addition of a likely chemosymbiotic bivalve taxon to the invertebrate fauna associated with this specimen provides more support for interpreting the type specimen of *Borealodon* as representing a sulfophilic-stage whale-fall community. Today's whale-fall communities advance through ecological stages that culminate in a sulfophilic stage where *Osedax* is present along with other invertebrate (although no other bone-boring) taxa [30,31]. Interestingly, Goedert & Squires [32] reported fossil chemosynthetic bivalves belonging to the vesicomyid genus *Calyptogena* from LACMIP locality 6295 (= LACMVP locality 5412), also located on the west side of Murdock Creek in Washington State (see type locality information), indicating that fossil whale-fall communities probably harbour richer associated invertebrate communities than more broadly appreciated.

Fossil cetacean skeletal material that comprise examples of chemosymbiotic fossil whale-falls have been described from Oligocene and Miocene localities along the North Pacific basin [11,12,33]. The presence of *Osedax* with clear taxonomic identification for the host skeleton (*Borealodon*, in this case) is unique for *Osedax* associations in the fossil record thus far, which includes a range of marine tetrapod host taxa from the Cretaceous to the Cenozoic from deep-sea rock deposits [34]. As Kiel *et al.* [11,12] proposed, *Osedax* itself may be a major contributor to an unknown (and potentially unknowable) component of the removal of skeletal material after death but prior to burial and incorporation into the geologic record.

Lastly, other authors have reported that during preparation of USNM 539939, five teeth of the shark *Somniosus gonzalezi* were discovered in the surrounding matrix [35]. These authors, as well as previous authors [11], speculated that the shark teeth may have been shed while scavenging the carcass. While we agree that it is possible, there is no evidence of scavenging directly preserved on the skeletal material of USNM 539939 itself, contra Kiel *et al.* [11].

## 6. Conclusion

*Borealodon osedax* represents a new lineage of toothed, stem mysticete crown-ward to mammalodontids and stem-ward to aetiocetids. *Borealodon* preserves elaborate, multi-cusped teeth with apical and occlusal wear reminiscent of *Salishicetus* and *Fucaia*. MicroCT scans of the inner ear indicate that *Borealodon* had a lower frequency limit of hearing similar to mammalodontids. The preservation of *Osedax* burrows on the type material of *Borealodon* is unique in that the host taxon now has a clear taxonomic identification. *Borealodon* offers more insights into the extent to which *Osedax* scavenging may remove skeletal material and more broadly impact the cetacean fossil record, as well as into the evolution of these deep-sea whale-fall communities overall.

## Supplementary Material

Figure S1

## Supplementary Material

Phylogenetic Analysis Matrix

## Data accessibility

The following information was supplied regarding data availability: Zenodo: doi:10.5281/zenodo.1340712. https://zenodo.org/record/1340712#.XLX6gC_MzOQ.
