# Supplementary figures and images for: Borealodon osedax, a new stem mysticete (Mammalia, Cetacea) from the Oligocene of Washington State and its implications for fossil whale-fall communities
Source: R Soc Open Sci. 2019 Jul 24;6(7):182168. doi: 10.1098/rsos.182168 (PMC6689636; doi:10.1098/rsos.182168)

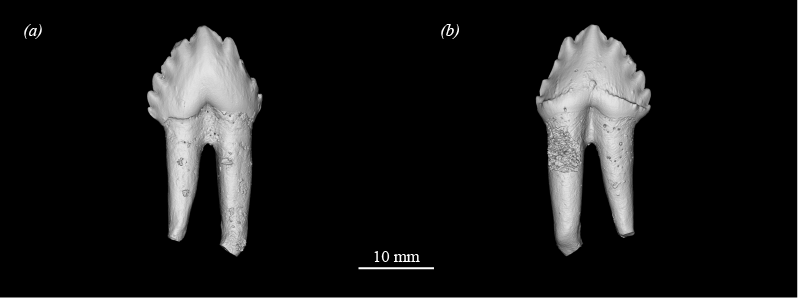

Supplement: Figure S1 [file rsos182168supp1.tif]
